# Long-Term Behavior of Defined Mixed Cultures of *Geobacter sulfurreducens* and *Shewanella oneidensis* in Bioelectrochemical Systems

**DOI:** 10.3389/fbioe.2019.00060

**Published:** 2019-03-27

**Authors:** Christina Engel, Florian Schattenberg, Katrin Dohnt, Uwe Schröder, Susann Müller, Rainer Krull

**Affiliations:** ^1^Institute of Biochemical Engineering, Technische Universität Braunschweig, Braunschweig, Germany; ^2^Braunschweig Centre of Systems Biology (BRICS), Technische Universität Braunschweig, Braunschweig, Germany; ^3^Working Group Flow Cytometry, Department of Environmental Microbiology, Centre for Environmental Research – UFZ, Leipzig, Germany; ^4^Institute of Environmental and Sustainable Chemistry, Technische Universität Braunschweig, Braunschweig, Germany

**Keywords:** bioelectrochemical systems, biofilm thickness, flow cytometry, *Geobacter sulfurreducens*, microbial electrolysis cell, mixed culture, planktonic, *Shewanella oneidensis*

## Abstract

This work aims to investigate the long-term behavior of interactions of electrochemically active bacteria in bioelectrochemical systems. The electrochemical performance and biofilm characteristics of pure cultures of *Geobacter sulfurreducens* and *Shewanella oneidensis* are being compared to a defined mixed culture of both organisms. While *S. oneidensis* pure cultures did not form cohesive and stable biofilms on graphite anodes and only yielded 0.034 ± 0.011 mA/cm^2^ as maximum current density by feeding of each 5 mM lactate and acetate, *G. sulfurreducens* pure cultures formed 69 μm thick, area-wide biofilms with 10 mM acetate as initial substrate concentration and yielded a current of 0.39 ± 0.09 mA/cm^2^. Compared to the latter, a defined mixed culture of both species was able to yield 38% higher maximum current densities of 0.54 ± 0.07 mA/cm^2^ with each 5 mM lactate and acetate. This increase in current density was associated with a likewise increased thickness of the anodic biofilm to approximately 93 μm. It was further investigated whether a sessile incorporation of *S. oneidensis* into the mixed culture biofilm, which has been reported previously for short-term experiments, is long-term stable. The results demonstrate that *S. oneidensis* was not stably incorporated into the biofilm; rather, the planktonic presence of *S. oneidensis* has a positive effect on the biofilm growth of *G. sulfurreducens* and thus on current production.

## Introduction

Bioelectrochemical systems (BESs) have gained great interest over the past few decades (Wang and Ren, 2013). In these systems electrodes serve either as an electron donor or as an electron acceptor to a special type of bacteria, which are termed “electrochemically active” (Lovley, 2006). Naturally these bacteria reduce insoluble minerals containing oxidized metal ions to gain energy (Lovley, 2006). Alternatively, they are also able to reduce anodes in microbial fuel cells (MFCs) or microbial electrolysis cells (MECs) (Logan, 2009; Wang and Ren, 2013).

Among the electrochemically active bacteria, *Geobacter sulfurreducens* and *Shewanella oneidensis* are most commonly studied. Especially *G. sulfurreducens* or its close relative *G. anodireducens* is well-known in bioelectrochemical research, since this bacterium is found highly enriched on anodic biofilms derived from municipal wastewater in MFCs or MECs, indicating its superior ability to perform extracellular electron transfer over other species (Lovley, 2006; Harnisch et al., 2011; Schmidt et al., 2017).

The two organisms differ in their metabolic abilities. *S. oneidensis* is a facultative anaerobe microorganism and is able to degrade a variety of organic acids (Venkateswaran et al., 1999; Ross et al., 2011; Chubiz and Marx, 2017). Under aerobic conditions, *S. oneidensis* is able to completely oxidize organic acids, however under anaerobic conditions the oxidation can only be achieved as far as acetate (Lovley, 2006). *G. sulfurreducens*, on the other hand, prefers acetate as carbon and electron source, which it mineralizes anaerobically to CO_2_ and H_2_O (Lovley, 2006). However, it has been shown that *G. sulfurreducens* can also degrade other organic acids like lactate and formate (Call and Logan, 2011; Speers and Reguera, 2012).

The transfer of electrons to the extracellular anode can be accomplished in two different ways, either biofilm mediated or based on soluble mediators (Babauta et al., 2012; Zhou et al., 2013). In both *G. sulfurreducens* and *S. oneidensis*, biofilm mediated electron transfer is catalyzed by a network of c-type cytochromes located in the periplasmic space, the outer membrane and the extracellular space and will occur if the bacteria are in contact to the electrode (Inoue et al., 2011; Ross et al., 2011; Liu and Bond, 2012; Santos et al., 2015). Maintaining a close proximity to the electrode facilitates electron transfer and can be accomplished by forming a biofilm on the anode (Dolch et al., 2014). *G. sulfurreducens* is a very good biofilm producer, often achieving biofilm thicknesses up to 160 μm (Leang et al., 2013). Cells in upper biofilm layers are hypothesized to establish contact by producing conductive pili, so called nanowires, which form a relay network together with extracellular c-type cytochromes through which electrons can be transferred directly to the electrode surface (Reguera et al., 2005; Ordóñez et al., 2016; Reguera, 2018). In mixed cultures derived from municipal wastewater, even thicker *G. sulfurreducens* based biofilms of up to 205 μm have been reported (Baudler et al., 2015). Further, mixed consortia have mainly been reported to produce higher power densities than pure cultures (Ishii et al., 2008; Logan, 2009), which indicates that cultivation in a mixed consortium can be advantageous. *S. oneidensis* on the other hand is only a poor biofilm producer and performs about 75% of its electron transfer based on soluble mediators (Marsili et al., 2008; Kotloski and Gralnick, 2013; Dolch et al., 2014). The mediators are redox molecules like flavins, which are reduced by planktonic *S. oneidensis* and reoxidized at the anode (Marsili et al., 2008; Kotloski and Gralnick, 2013). In general, c-type cytochrome mediated electron transfer is advantageous to soluble mediator based transfer, since the close proximity to the anode allows for fast electron transfer, while the soluble mediator based transfer is limited by the continuous need to produce new electron shuttles in the case of washout (Inoue et al., 2011; Dolch et al., 2014).

Often, research in BESs is done with biofilms derived from municipal wastewater inocula, in which *G. sulfurreducens* or other *Geobacter* species are enriched and predominant in the anodic biofilm (Logan, 2009). These systems are easy to handle and insensitive to intruding oxygen, and the conclusion that *G. sulfurreducens* or its close relative *G. anodireducens* is responsible for the observed bioelectrochemical behavior is reasonable because of its great abundance (Logan, 2009; Sun et al., 2014). But the individual functions of the other organisms in the system cannot be easily determined and related to a single species (Logan, 2009). Therefore, a systematic approach applied here is the use of defined mixed cultures, as in these systems the complexity of an undefined, randomly generated wastewater based system is reduced to a defined number of bacterial species. Thus, the influence of a specific species on a *G. sulfurreducens* based biofilm can be determined in a more controlled way. For example Qu et al. (2012) have shown that the combined cultivation of *E. coli* and *G. sulfurreducens* enables *G. sulfurreducens* to produce current in a single chamber MFC setup, as *E. coli* consumes oxygen intruding from the air cathode used in this study. In other investigations, Venkataraman et al. (2011) could observe higher current densities in a mixed culture of *Pseudomonas aeruginosa* and *Enterobacter aerogenes* compared to respective pure cultures, and Rosenbaum et al. (2011) achieved the conversion of glucose to current by a combined cultivation of *S. oneidensis* with *Lactococcus lactis*.

The specific interactions of *G. sulfurreducens* and *S. oneidensis* have been studied before. A combined cultivation resulted in higher amounts of sessile cells of both species on graphite felt in MFCs operated in galvanostatic mode (Dolch et al., 2014). The amount of sessile cells of *S. oneidensis* was even increased by 11-fold compared to *S. oneidensis* pure cultures (Dolch et al., 2014). However, experiments only lasted <2 days (Dolch et al., 2014), so it was not investigated whether the incorporation of *S. oneidensis* was stable or whether *G. sulfurreducens* eventually dislodges *S. oneidensis* from the electrode. In a following study, the amount of *S. oneidensis* and *G. sulfurreducens* was studied together with *G. metallireducens* on activated carbon cloth at an anode potential of 0 V (Prokhorova et al., 2017). *G. sulfurreducens* was found to be predominant (ca. 95%) in the biofilm, but *S. oneidensis* (ca. 5%) and *G. metallireducens* were also incorporated and were able to grow within the mixed culture biofilm over a course of 7 days (Prokhorova et al., 2017). Reactors used in that study were not air-tight, however, which supports growth of *S. oneidensis* (Teravest et al., 2014).

Therefore the aim of this study is to test the positive effects seen by the previous studies (Dolch et al., 2014; Prokhorova et al., 2017) in an air-tight and anaerobically operated MEC setup and to observe the long-term behavior of the defined mixed culture of *G. sulfurreducens* and *S. oneidensis*. Further, the stable incorporation of *S. oneidensis* in the mixed culture biofilm is investigated after 44 days with intermediate removal of planktonic cells, which allows relating observed effects to biofilm associated cells alone.

## Materials and Methods

### Bacterial Strains and Cultivation Medium

*Geobacter sulfurreducens* PCA (DSMZ 12127) and *Shewanella oneidensis* MR1 (ATCC 700550) were used in this study.

*G. sulfurreducens* was cultivated in a sterile minimal medium containing 10 mM acetate as carbon source and electron donor and 40 mM fumarate as electron acceptor. Other components of the medium were (per liter of deionized water) (Balch et al., 1979; Kim et al., 2005): 0.31 g of NH_4_Cl, 0.13 g of KCl, 2.69 g of NaH_2_PO_4_ × H_2_O, 4.33 g of Na_2_HPO_4_, 12.5 mL of trace mineral solution (containing per liter: 1.5 g of nitrilotriacetic acid, 3 g of MgSO_4_ × 7 H_2_O, 0.5 g of MnSO_4_ × 2 H_2_O, 1 g of NaCl, 0.1 g of FeSO_4_ × 7 H_2_O, 0.1 g of CoCl^−2^, 0.1 g of CaCl_2_ × 2 H_2_O, 0.13 g of ZnSO_4_, 0.1 g of CuSO_4_ × H_2_O, 0.1 g of AlK(SO_4_)_2_, 0.1 g of H_3_BO_3_, 0.01 g of Na_2_MoO_4_ × 2 H_2_O, 0.3 mg of Na_2_SeO_3_ × 5 H_2_O and 0.03 g of NiCl_2_ × 6 H_2_O) and 12.5 mL of vitamin solution (containing per liter: 2 mg of biotin, 2 mg of folic acid, 10 mg of pyridoxine hydrochloride, 5 mg of thiamine hydrochloride, 5 mg of riboflavin, 5 mg nicotinic acid, 5 mg DL-calcium pantothenate, 0.1 mg of vitamin B12, 5 mg of p-aminobenzoic acid, and 5 mg of lipoic acid). All chemicals were purchased from Sigma-Aldrich, Carl Roth or Merck at technical grade.

Medium for precultures was prepared in sterilized serum bottles (125 mL, Fisher Scientific, Hampton, New Hampshire, USA) and gassed with N_2_ for a minimum of 20 min to obtain anaerobic conditions. Subsequently 20% (v/v) of a consisting *G. sulfurreducens* culture (early stationary phase, OD_600_ of ~0.5) was added as inoculum and serum bottles were sealed with butyl rubber stoppers. The preculture suspension was added directly as centrifugation of cells resulted in a thin cell film distributed over the entire side of centrifugation tubes that did not allow reproducible inoculation. Cells were incubated at 180 min^−1^ (25 mm orbital diameter) and 30°C for about 24 h. For bioelectrochemical experiments, fumarate was omitted from the medium, so that the electrode served as the sole electron acceptor. Medium was prepared in bioelectrochemical cells (see 2.2), gassed with N_2_ and cultivations were inoculated with cell suspension from a preculture (early stationary phase, OD_600_ of ~0.5). The added volume of the preculture was chosen so that an initial optical density (600 nm) of 0.1 was achieved.

*S. oneidensis* was cultivated aerobically in baffled shake flasks (250 mL shake flask, 4 baffles, 25 mL filling quantity) in minimal medium containing 10 mM of lactate as carbon source and electron donor while oxygen served as the electron acceptor. Other components of the medium were (per liter of deionized water) (Hau et al., 2008): 0.225 g of K_2_HPO_4_, 0.225 g of KH_2_PO_4_, 0.46 g of NaCl, 0.225 g of (NH_4_)_2_SO_4_, 0.117 g of MgSO_4_ and 12.5 mL each of the trace mineral solution and vitamin solution also used for *G. sulfurreducens*. Cells were cultivated at 180 min^−1^ (25 mm orbital diameter) and 30°C. For bioelectrochemical experiments, medium was prepared within the bioelectrochemical cells and gassed with N_2_ to obtain anaerobic conditions. 10 mM of lactate were replaced by 5 mM of acetate and 5 mM of lactate. Cultivations were inoculated with cells from aerobic precultures centrifuged for removal of old medium at 5,900 g for 10 min at 4°C to result in an optical density (600 nm) of 0.1. 1 mL of the prepared medium from bioelectrochemical cells was used to resuspend cell pellets.

For defined mixed cultures, the medium for *G. sulfurreducens* was used, however, instead of 10 mM acetate, 5 mM of acetate and 5 mM of lactate were used as carbon and electron source. Bioelectrochemical cells were inoculated with both cell types to result in an optical density (600 nm) of 0.1 for each organism.

### Electrochemical Setup and Techniques

**Figure 1:**
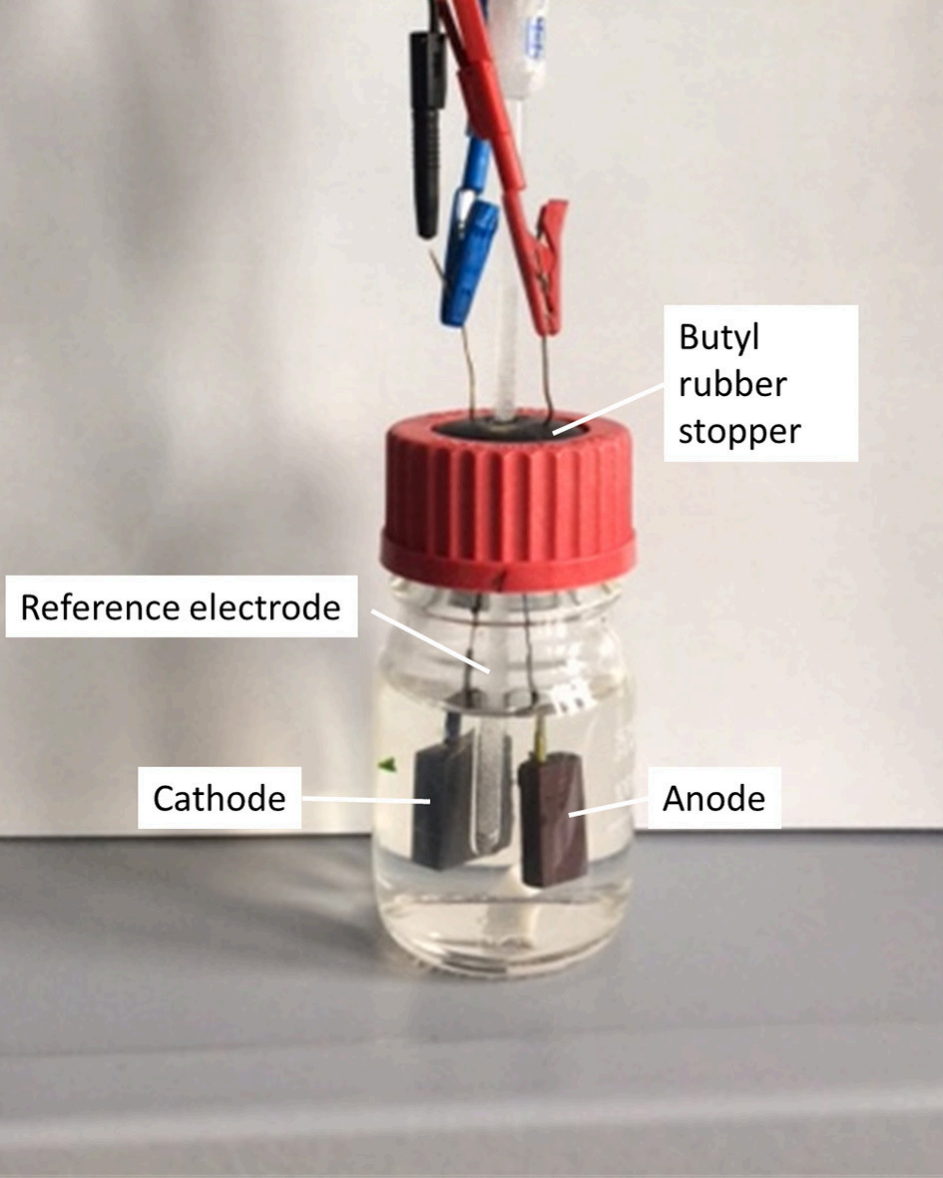
MEC-setup used for electrochemical experiments.

Cultivations were carried out in half cell setups (one-chamber, three-electrode arrangement) under potentiostatic control. The bioelectrochemical cell is depicted in Figure 1 and was composed of a 100 mL flask sealed with a butyl rubber stopper. A 5 mm diameter hole was milled in the stopper to allow the insertion of the Ag/AgCl sat. KCl reference electrode (SE11, Meinsberger, Waldheim, Germany). Graphite plates of 1 × 2.5 × 0.5 and 2 × 2.5 × 0.5 cm, which were ground with 240-grit sandpaper and rinsed with deionized water for cleaning, served as working and counter electrode, respectively. They were connected with silver-coated copper wires (0.6 mm diameter) which were transferred through the butyl rubber stopper to allow for an oxygen tight connectivity. Copper wires were isolated with shrink-on tubes to prevent electrochemical reactions with the medium. The flasks contained a stirring bar (2.5 cm length) to allow for mixing on a magnetic stirrer at 300 min^−1^.

After inoculation, the working electrode potential was set to 0.2 V_Ag/AgCl_ in chronoamperometric mode by means of a potentiostat (MPG2, BioLogic Science Instruments, Seyssinet-Pariset, France). The reduction of protons occurred at the counter electrode. Once current production subsided, the medium was replaced by fresh medium for a second and third cycle to provide the cells with more electron donor (5 mM lactate and 5 mM acetate) and selecting for sessile cells. Hereby, medium was prepared as described above in a new flask and gassed with N_2_ for 20 min. Subsequently, the butyl rubber stopper containing the electrodes and the electrochemically active biofilm was transferred into the fresh medium. At the beginning and end of each cycle, a sample was taken to allow for the determination of acetate and lactate concentrations. Both were measured using an HPLC system (LaChrom Elite^®^, Hitachi, Tokyo, Japan) equipped with an Aminex^®^ HPX-87H column, Bio-Rad Laboratories, Hercules, California, USA) as the stationary phase and 12.5 mM H_2_SO_4_ as mobile phase. Lactate was determined at 0.5 mL/min and 45°C with a retention time of approximately 15.6 min, while acetate was determined at 0.3 mL/min and 25°C with a retention time of approximately 33 min. Detection was performed using a refractive index detector.

From the charge quantity measured during chronoamperometry and the substrate consumption as determined by HPLC, the coulombic efficiency (CE) was calculated as described previously by others (Oh et al., 2004). During the third cycle and before current subsided, experiments were terminated for confocal laser scanning microscopy and cytometric analysis. All experiments were performed in biological triplicates.

### Confocal Laser Scanning Microscopy (CLSM)

After termination of experiments, biofilms grown on graphite anodes were analyzed by confocal laser scanning microscopy (CLSM, C2+, Nikon, Düsseldorf, Germany). Cells were stained with a solution containing 113 μM acridine orange and 60 μM propidium iodide for 5 min as adapted from Brown et al. (2005) and Speers and Reguera (2012). Subsequently the dying solution was removed and anodes were placed in petri dishes and overlaid with a solution containing 0.31 g of NH_4_Cl, 0.13 g of KCl, 2.69 g of NaH_2_PO_4_ × H_2_O and 4.33 g of Na_2_HPO_4_ per liter of deionized water to prevent the biofilm from drying. Immediately following this, CLSM measurements were performed using a 488 and a 561 nm laser respectively and an air objective (10x).

For each biofilm, 5 images were recorded from different spots on the electrode. The volume (*V*) and covered electrode area (*A*_*b*_) were determined from the fluorescence signal using NIS- Elements Advanced Research software (Nikon, Düsseldorf, Germany) whereby a fluorescence intensity of 8% or higher was considered to be biofilm. The average biofilm thickness (*d*) was calculated from this data with $d=\;\frac{V}{A_{b}}$.

### Flow Cytometry Analysis

#### Cell Fixation

During the third cultivation cycle, biofilms, and planktonic cells were harvested and fixed for subsequent flow cytometry analysis as described previously (Zimmermann et al., 2016). Biofilms were scraped off the electrode using a metal inoculation loop and resuspended in PBS solution (8 g/L NaCl, 0.2 g/L KCl, 1.44 g/L Na_2_HPO_4_, 0.24 g/L KH_2_PO_4_ by vortexing. Both planktonic and resuspended biofilm cells were then centrifuged (3,200 g, 4°C, 10 min) and washed two times with 2 mL PBS. Subsequently, pellets were resuspended in 4 mL of a 2% paraformaldehyde solution [1 mL of 8% (w/w) paraformaldehyde + 3 mL of PBS] and incubated at 4°C for 30 min. After a final centrifugation, pellets were resuspended and stored in 4 mL 70% ethanol at −20°C.

#### Cell Staining

The staining procedure was done according to Koch et al. (2013). The optical density (OD) of the well-mixed PFA (2%) / EtOH (70%) fixed samples was measured (d_⋌700nm_ = 0.5 cm) and adjusted to 0.035 with PBS (6 mM Na_2_HPO_4_, 1.8 mM NaH_2_PO_4_, 145 mM NaCl with bi-distilled H_2_O, pH = 7). After centrifugation of 2 mL of this solution for 10 min at 4°C and 3,200 g the supernatant was discarded. The cell-pellet was resuspended in 1 mL of permeabilization buffer (0.1 M citric acid, 4.1 mM Tween 20, bi-distilled water) and incubated for 20 min at room temperature. After a further centrifugation step the supernatant was discarded and the cells were resuspended in 2 ml DNA staining solution [0.24 μM 4′,6-di-amidino-2-phenyl-indole (DAPI, Sigma-Aldrich, St. Louis, USA), in 417 mM Na_2_HPO_4_/NaH_2_PO_4_ buffer (289 mM Na_2_HPO_4_, 128 mM NaH_2_PO_4_ with bi-distilled H_2_O, pH = 7)] for subsequent staining overnight at room temperature until cytometric measurement. A biological standard [*Escherichia coli* BL21 (DE3), stationary phase of growth curve (16 h), fixed with PFA (2%) / EtOH (70%)] was stained as above.

#### Flow Cytometry

The MoFlo Legacy cell sorter (Beckman-Coulter, Brea, California, USA) equipped with two lasers was used for generating cytometric data. The blue laser Genesis MX488-500 STM OPS (Coherent, Santa Clara, California, USA) (488 nm, 400 mW) was used to measure the forward scatter (FSC; bandpass filter 488 nm ± 5 nm, neutral density filter 1.9) which is an optical characteristic containing information related to cell size and the side scatter (SSC; bandpass filter 488 ± 5 nm, neutral density filter 1.9, trigger signal), an optical characteristic containing information related to cell density. The UV laser Xcyte CY-355-150 (Lumentum, Milpitas, California, USA) (355 nm, 150 mW) was used for exciting the DAPI fluorescence (bandpass filter 450 ± 32.5 nm), an optical characteristic that is used for quantification of cellular DNA-content. Photomultiplier tubes were purchased from Hamamatsu Photonics (Models R928 and R3896; Hamamatsu City, Japan). The fluidic system was run at 56 psi (3.86 bar) with sample overpressure at maximum 0.3 psi (0.02 bar) and a 70 μm nozzle. The sheath fluid was composed of 10x Sheath buffer (19 mM KH_2_PO_4_, 38 mM KCl, 166 mM Na_2_HPO_4_, 1.39 M NaCl with bi-distilled H_2_O) diluted with 0.1 μm filtrated bi-distilled H_2_O to a 0.2x working solution. Prior to all measurements, daily calibration of the instrument was performed linearly with 1 μm blue fluorescent beads [FluoSpheres F8815 (350/440), lot no.: 69A1-1] and 2 μm yellow-green fluorescent beads [FluoSpheres F-8827 (505/515), lot no.: 1717426], both from Molecular Probes (Eugene, Oregon, USA). Blue fluorescent beads [0.5 μm and 1 μm, both Fluoresbrite BB Carboxylate microspheres, (360/407), lot no.: 552744 and 499344, PolyScience, Niles, Illinois, USA] were used for calibration of logarithmic scale and added to each sample for measurement stability. A biological standard [*Escherichia coli* BL21 (DE3)] was measured as a biological adjustment. The stained samples were filtered using 50 μm CellTrics filter (Sysmex Partec GmbH, Görlitz, Germany) before measurement to prevent clogging of the cytometer nozzle. Every sample was measured at a maximum speed of 3,000 events/s.

Cytometric data acquisition and visualization was performed using Summit Ver. 4.3 (Beckman-Coulter, Brea, California, USA) and FlowJo Ver. 10 (FlowJo, LLC, Oregon, USA) using the dot-plot option FSC (cell size) against DAPI-fluorescence. A parent gate for measurement has been created with Summit Ver. 4.3 comprising all stained cells and excluding noise and beads (Koch et al., 2013, Figure S1). The measurement was done until 250,000 cells within this parent gate were detected. Due to the absence of a flurry distribution it was ensured that cells were well-dispersed and no cell aggregates were present (Lambrecht et al., 2018). A cell gate bearing to the already defined parent gate without noise and beads in Summit has been defined in FlowJo Ver. 10, depicted with an exemplary sample (Figure S2). In this cell gate the gate template consisting of the 2 gates *S. oneidensis* and *G. sulfurreducens* was created. Since the DAPI fluorescence in defined mixed culture samples was overall higher than in the pure culture samples, the cell gates had to be adjusted slightly and the results should therefore only be compared qualitatively.

The flow cytometric data files are deposited in the FlowRepository database (www.flowrepository.org) with the repository ID FR-FCM-ZYJR.

## Results

### Pure Cultures of *G. sulfurreducens* and *S. oneidensis*

**Figure 2:**
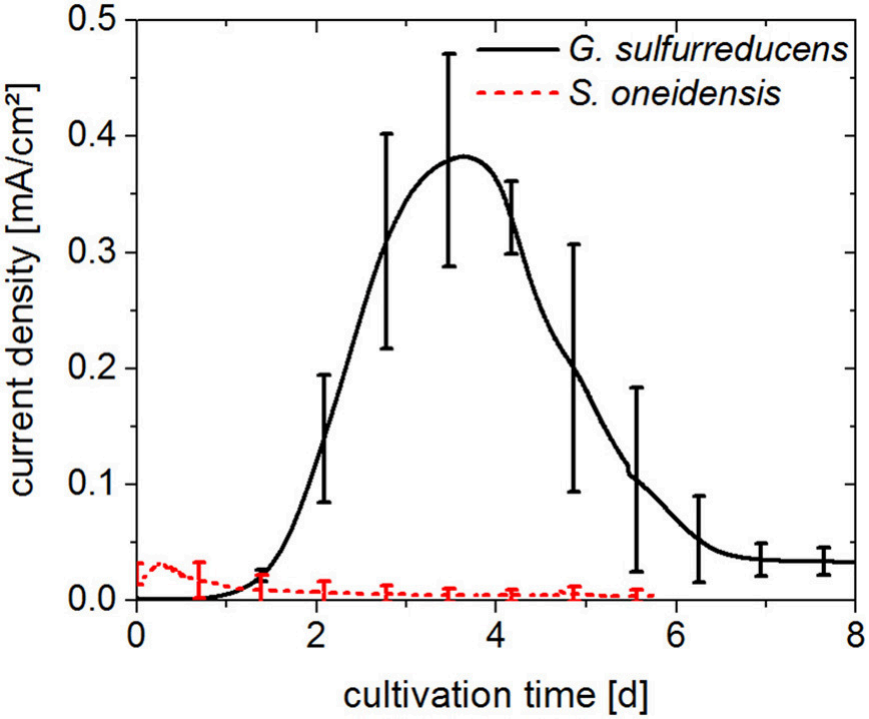
Chronoamperograms (first cultivation cycle) from pure cultures of *G. sulfurreducens* fed with 10 mM acetate (solid line) and *S. oneidensis* fed with 5 mM acetate and 5 mM lactate (dotted line). Anode potential: 0.2 V_Ag/AgCl_. Error bars represent standard deviation of biological triplicates. Samples were taken at the beginning and end of experiments.

*G. sulfurreducens* and *S. oneidensis* were first cultivated separately, in individual electrochemical cells. The resulting chronoamperograms of the first cultivation cycle are shown in Figure 2. With *G. sulfurreducens*, current density increased significantly 1 day after inoculation and reached its maximum value of 0.39 ± 0.09 mA/cm^2^ after 4 days of cultivation (three biological replicates). Subsequently, current density decreased within 8 days of cultivation. During this time, acetate was consumed almost completely as revealed by a sample taken at the end of the first cultivation cycle. There were no further samples taken during the course of the experiment to avoid disturbance of the cultivation e.g. by intruding oxygen. The CE for this cultivation was determined to be around 100%. Exchange of medium resulted in a second and third chronoamperometric cycle, which revealed similar maximum current densities (0.43 ± 0.05 and 0.35 ± 0.06 mA/cm^2^, respectively).

*S. oneidensis* cultures already reached their maximum current density of 0.034 ± 0.011 mA/cm^2^ within the first 12 h of the cultivation, after which time current rapidly decreased to values close to zero. Sixty percent of the lactate provided in the medium was consumed; however acetate was present at a concentration 30% higher than the initial concentration, as was seen in samples taken at the end of the first cultivation cycle after 6 days. The CE for the cultivation of *S. oneidensis* was around 10%.

**Figure 3:**
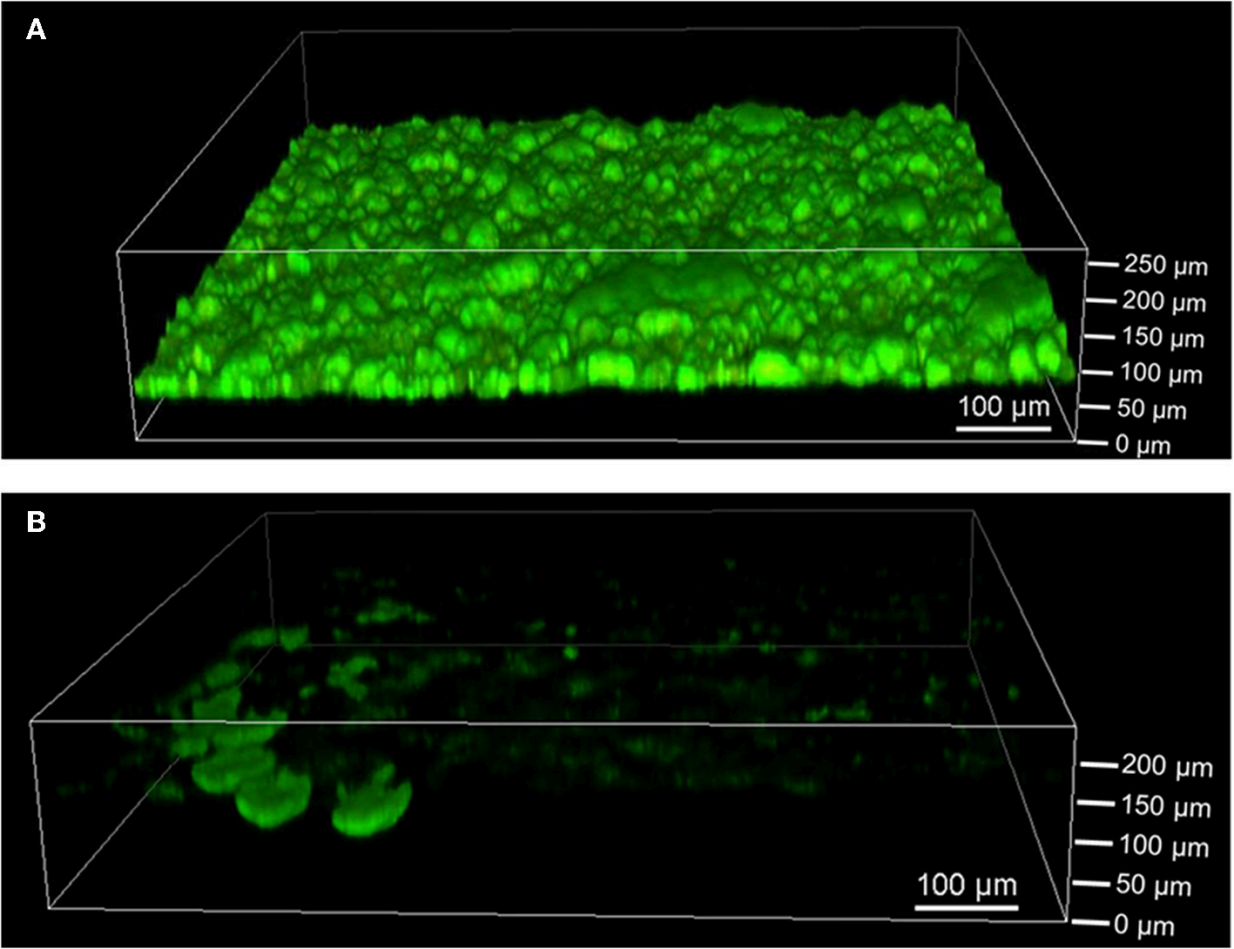
Confocal laser scanning microscope images of **(A)** *G. sulfurreducens* and **(B)** *S. oneidensis* pure cultures on graphite anodes stained with acridine orange (green) and propidium iodide (red) for live/dead staining. Five images were taken per experiment and experiments were performed in biological triplicates.

The extent of biofilm formation was analyzed at the end of the bioelectrochemical experiments by means of CLSM. Figure 3A shows a representative image of *G. sulfurreducens*. *G. sulfurreducens* formed cohesive biofilms of 69 ± 18 μm thickness on the graphite anodes. *S. oneidensis*, however, was barely detected (Figure 3B). Only on very few areas of the electrode did *S. oneidensis* form cell spots, which reached a thickness of up to 40 μm. A staining was performed for biofilm visualization, depicting cells with an intact membrane in green color, while cells with an impaired membrane appear red. It shows that both cultures revealed highly vital biofilms.

### Defined Mixed Culture

**Figure 4:**
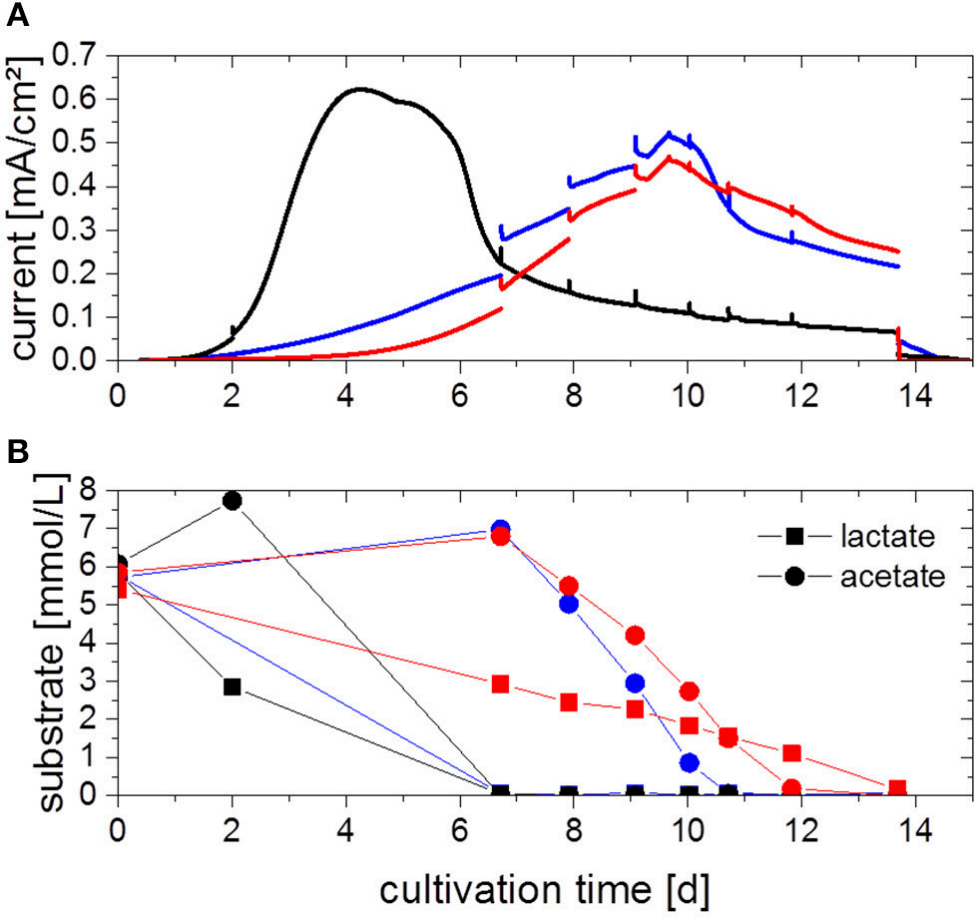
**(A)** Chronoamperograms (first cultivation cycle) from defined mixed culture of *G. sulfurreducens* and *S. oneidensis* fed with 5 mM acetate and 5 mM lactate with **(B)** corresponding acetate and lactate concentration as determined by HPLC analysis. Colors refer to three parallel replicates. Anode potential: 0.2 V_Ag/AgCl_. Current spikes are due to sample taking.

To investigate the potential benefits of a combined cultivation, both organisms were cultivated in electrochemical cells under the same process conditions as the pure cultures. The chronoamperograms (first cultivation cycle) of three parallel experiments are shown in Figure 4A. Two different electrochemical behaviors can be observed, one in which the current density increases within 4 days (black line, Figure 4A) similar to the *G. sulfurreducens* pure cultures (cp. Figure 2), and one in which the current density only reaches its maximum value after 10 days (blue and red lines, Figure 4A). The maximum current density achieved in these experiments is 0.54 ± 0.07 mA/cm^2^ and thus 38% higher compared to *G. sulfurreducens* pure cultures.

For all triplicates, the concentration profiles of the substrates acetate and lactate are depicted in Figure 4B. The trends are different for each replicate, although all replicates show an immediate start of a gradual degradation of lactate, while primarily an increase in acetate concentration is observed, followed likewise by its degradation. In all cases substrates were consumed completely at the time when current subsided, in the case of the faster experiment (black line, Figure 4A) after 7 days, in the other two cases after 11 and 14 days, respectively. From this data, a CE of about 120% could be determined. Biofilms were also characterized by cyclic voltammetry (CV) (Fricke et al., 2008, see Figure S3), which revealed that the defined mixed culture is showing the same electrochemical behavior as the *G. sulfurreducens* pure culture.

Medium was exchanged for a second and third chronoamperometric cycle, whereby all planktonic cells were removed to investigate the effect of only sessile cells. Maximum current decreased compared to the first cycle and only values of 0.30 ± 0.04 and 0.24 ± 0.05 mA/cm^2^, respectively, could be obtained.

**Figure 5:**
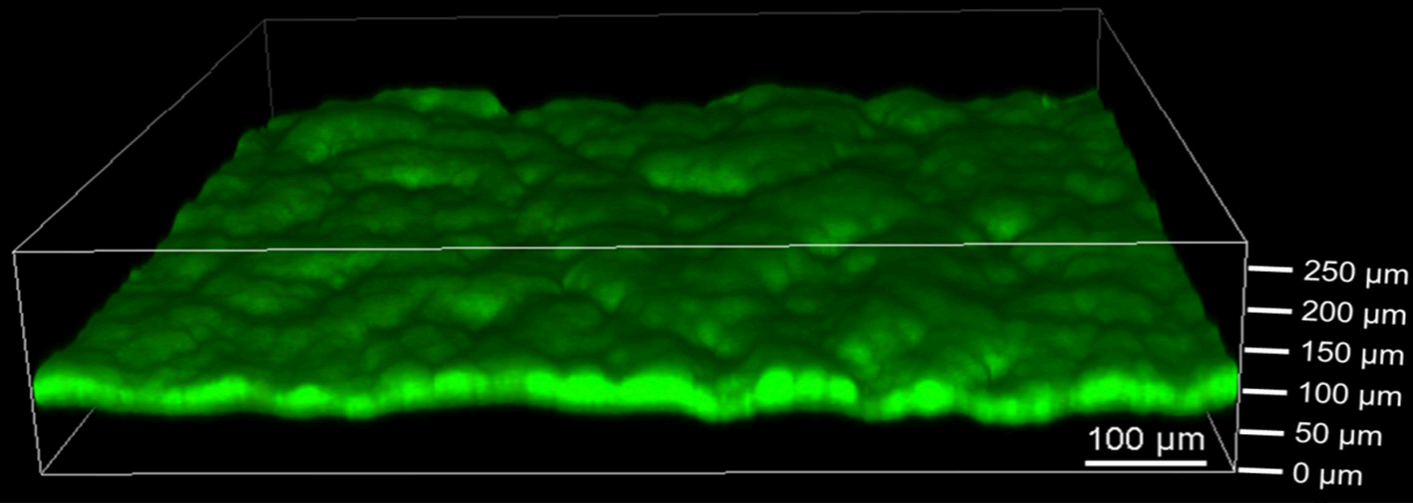
Confocal laser scanning microscope image of defined mixed culture of *G. sulfurreducens* and *S. oneidensis* on graphite anode stained with acridine orange (green) and propidium iodide (red) for live/dead staining. Five images were taken per experiment and experiments were performed in biological triplicates.

The defined mixed culture biofilms were also analyzed by CLSM as depicted in Figure 5. Cohesive biofilms with a thickness of 93 ± 8 μm formed on the anodes. Thus, *G. sulfurreducens*/*S. oneidensis* defined mixed culture biofilms are about 35% thicker than *G. sulfurreducens* pure culture biofilms. Like the pure cultures, the biofilms showed high cell viability. Defined mixed culture experiments that were analyzed earlier revealed that the biofilm thickness is already achieved within the first cultivation cycle (data not shown).

**Figure 6:**
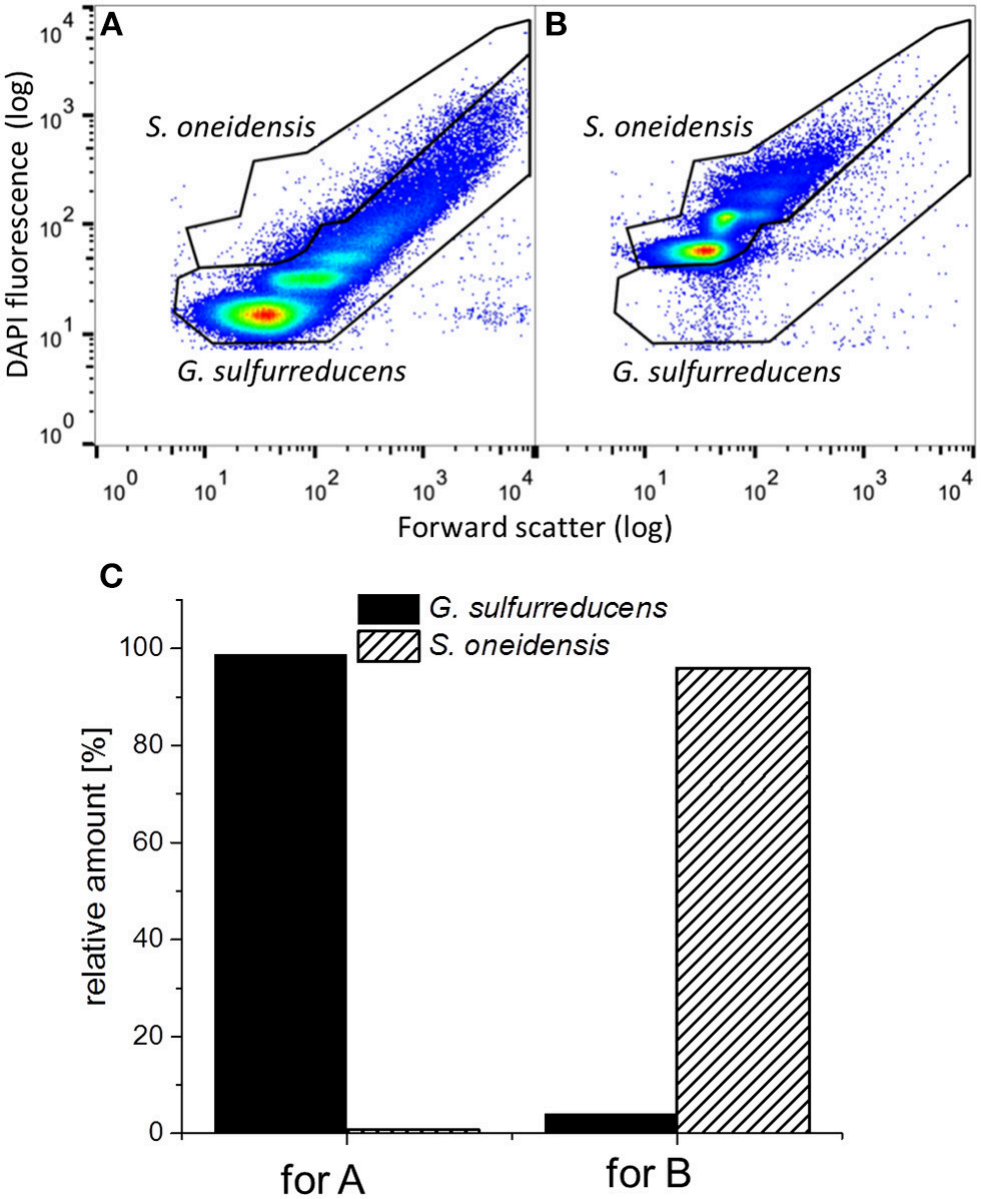
Representative dot plots from **(A)** a *G. sulfurreducens* pure culture biofilm and **(B)** a planktonic *S. oneidensis* pure culture and **(C)** the thereof determined relative amount of cell counts in the *G. sulfurreducens* gate (filled) and the *S. oneidensis* gate (diagonally ruled).

To determine the distribution of *G. sulfurreducens* and *S. oneidensis* in the defined mixed culture at the end of the third cycle, samples from both biofilm and planktonic cells were analyzed by flow cytometry, which is a useful technique to show distributions among single cells, even for electrochemically active cells (Koch et al., 2014). Figure 6 shows representative dot plots from pure cultures of *G. sulfurreducens* and *S. oneidensis* and the distribution of each cell type between their cell gates, which represent proliferation states within a population (Müller, 2007). For *G. sulfurreducens* cells, a pure culture biofilm sample was used (cp. Figure 6A). For *S. oneidensis*, planktonic cells had to be measured, since no sufficient amount of biofilm cells could be obtained from pure cultures of *S. oneidensis* (cp. Figure 6B). Figure 6C shows the distribution of each species determined from the number of cell counts in each cell gate. It can be seen that in both samples a small number of cells scattered into the opposite species' cell gate, thus creating a falsely positive signal, which was regarded in the evaluation of cell numbers per gate when both species were grown together in the electrochemical cell.

**Figure 7:**
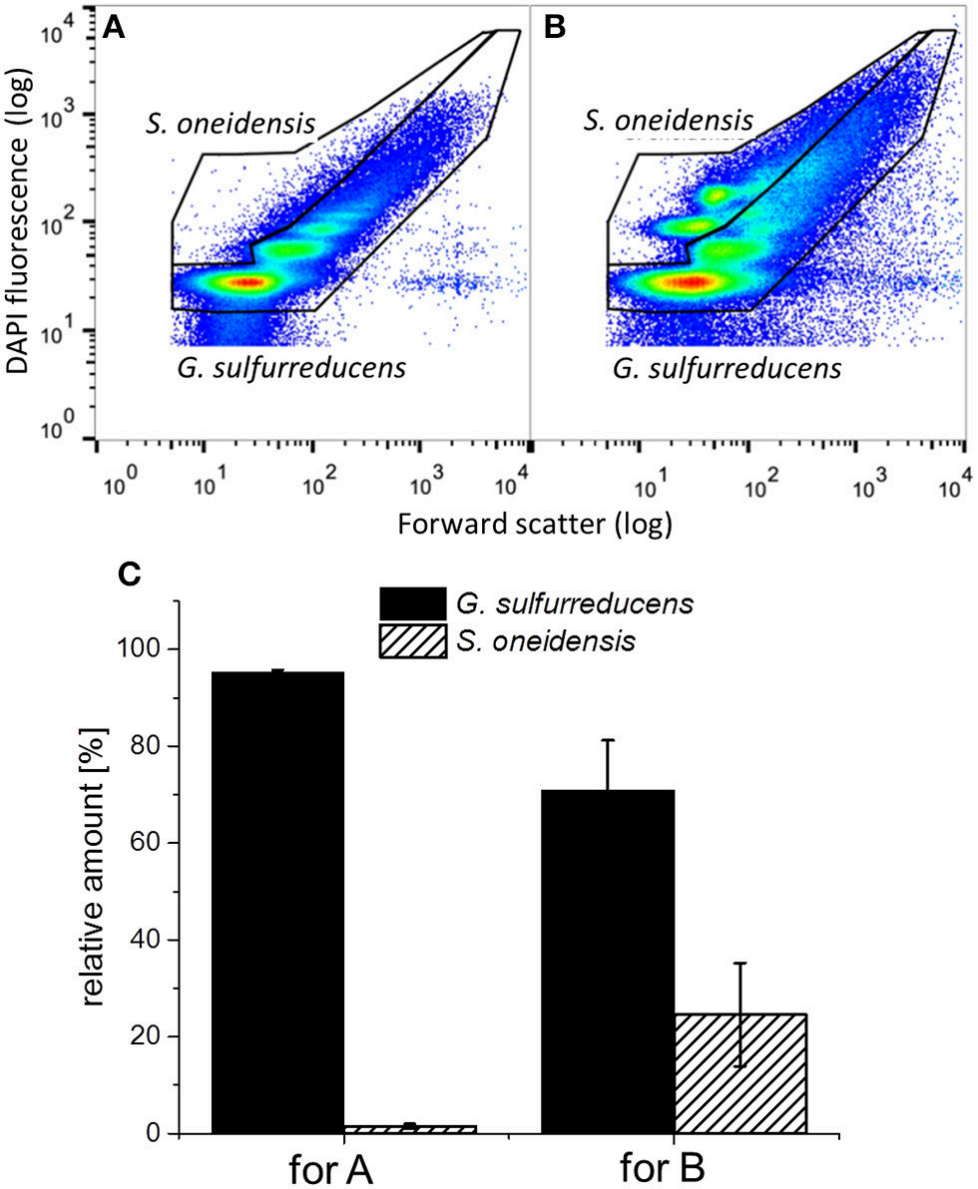
Representative dot plots from **(A)** the biofilm and **(B)** planktonic cells of a *G. sulfurreducens*/*S. oneidensis* defined mixed culture and **(C)** the thereof determined relative amount of cells in the *G. sulfurreducens* gate (filled) and the *S. oneidensis* gate (diagonally ruled). Error bars in C represent standard deviation from biological triplicates.

Figure 7 shows representative dot plots of biofilm (cp. Figure 7A) and planktonic cells (cp. Figure 7B) of a *G. sulfurreducens/S. oneidensis* defined mixed culture. The differentiation between the two cell types can be clearly identified. In Figure 7C the thereof calculated distribution of the two cell types is depicted. The amount of *S. oneidensis* detected within the biofilm was about 2% (column A), which is not substantially higher than the falsely positive amount of *S. oneidensis* found in a *G. sulfurreducens* pure culture biofilm (1%) (cp. with column A from Figure 6C). From column B of Figure 7C it can be seen that *S. oneidensis* cells are only found planktonically at a quantity of 25%, while 75% of planktonic cells are *G. sulfurreducens*.

## Discussion

### Pure Cultures of *G. sulfurreducens* and *S. oneidensis*

In electrochemical *G. sulfurreducens* pure cultures 10 mM acetate were consumed to result in current and 0.39 ± 0.09 mA/cm^2^ were observed as maximum current density. This value is in accordance with current densities observed for *G. sulfurreducens* pure cultures in the literature. For example Zhu et al. observed approximately 0.57 mA/cm^2^ on graphite poised to 0.3 V_Ag/AgCl_ with 10 mM acetate (Zhu et al., 2012).

The CE of approximately 100% observed with *G. sulfurreducens* is also in accordance with the observations of other authors; for instance Bond and Lovley (2003) have seen CEs of 95% with *G. sulfurreducens* with acetate as electron donor. This indicates viable and metabolically active cells.

In contrast, electrochemical *S. oneidensis* pure cultures merely yielded a ten times lower maximum current density of 0.034 ± 0.011 mA/cm^2^ and only lactate was consumed in these cultivations. This is in accordance with the inability of *S. oneidensis* to degrade acetate under anaerobic conditions (Lovley, 2006). Similar maximum current densities of 0.02 mA/cm^2^ with 20 mM lactate were observed by Teravest and Angenent (2014) on graphite paper. The CE of approximately 10% observed in this study is low compared to G. sulfurreducens, but similar CEs have been reported by others (Rosenbaum et al., 2011). The electrons unaccounted for were probably used to form H_2_, a process that was reported for *S. oneidensis* by Meshulam-Simon et al. (2007) for electron acceptor limiting conditions. Another possibility would be the consumption of oxygen leaking into the MEC. However, oxygen leakage can only have been minor as *G. sulfurreducens*, which reacts more sensitive to oxygen intrusion (Rosenbaum and Franks, 2014), was able to grow and produce current in the setup used.

CLSM analysis revealed *G. sulfurreducens* biofilms of 69 ± 18 μm, while only single cell spots of *S. oneidensis* were found on graphite anodes. The insufficient biofilm formation abilities of *S. oneidensis* compared to *G. sulfurreducens* have also been observed by others (Dolch et al., 2014). The poor biofilm is limiting the c-type cytochrome based electron transfer of *S. oneidensis*, which explains the much lower current densities seen with this bacterium compared to *G. sulfurreducens*. *S. oneidensis* alternatively uses flavins or other soluble mediators for electron transfer (Marsili et al., 2008). Kotloski and Gralnick determined 75% of *S. oneidensis*' electron transfer happens via flavins (Kotloski and Gralnick, 2013). These mediators have to be produced by the cells continuously and the metabolic burden of their production has yet to be determined (Kotloski and Gralnick, 2013). The ability of *G. sulfurreducens* to form thick and conductive biofilms is advantageous toward *S. oneidensis*, since electrons can be transferred directly and fast, mainly by means of conductive nanowires even from distances a few hundred micrometers off the electrode (Malvankar and Lovley, 2014).

The data clearly shows the greater potential of *G. sulfurreducens* as a ten times better current producing microorganism as opposed to *S. oneidensis*. However, *G. sulfurreducens* is limited in the amount of substrates it can consume, whereas *S. oneidensis* can use a broader range of organic acids, which it can degrade to acetate (Lovley, 2006). Since *G. sulfurreducens* is anaerobic, the cultivation of this bacterium is challenging, as small amounts of oxygen intruding into the system can already prevent the cells from using an anode as terminal electron acceptor (Caccavo et al., 1994; Rosenbaum and Franks, 2014). A combined cultivation including both organisms in oxygen-leaking systems was shown to be beneficial for each organism at a short time-scale (Dolch et al., 2014; Prokhorova et al., 2017). It remains unclear, however, if this benefit comes from planktonic cells or cells incorporated into the biofilm and whether this effect is stable on a longer time-scale. Therefore, a cultivation of *S. oneidensis* and *G. sulfurreducens* as defined mixed culture was performed for a longer time period with intermediate removal of planktonic cells.

### Defined Mixed Culture

Chronoamperograms from defined mixed cultures of *G. sulfurreducens* and *S. oneidensis* revealed two different electrochemical behaviors. Similarly, the concentration trends of acetate and lactate were different for each replicate. One possible explanation for this is that lactate degradation by *S. oneidensis* was insufficient and was to a certain extend carried out by *G. sulfurreducens*. The possibility of lactate consumption directly by *G. sulfurreducens* was reported before by others (Call and Logan, 2011). It was found that a single base pare mutation in a transcriptional regulator is responsible for the ability of lactate degradation (Summers et al., 2012). The spontaneity of this mutation could be the reason for the unreproducible lactate degradation and current production. Lactate consumption was found to result in an excretion of acetate due to an excess of carbon flow in the direction of acetyl-CoA (Segura et al., 2008; Speers and Reguera, 2012) and suggests that acetate uptake is inhibited in the presence of lactate. This corresponds with the observation of an acetate secretion here prior to acetate consumption. Experiments were performed under the assumption that lactate degradation by *S. oneidensis* would occur faster than possible lactate degradation by *G. sulfurreducens*. However, the results indicate an opposite behavior, suggesting that under anaerobic conditions, lactate consumption by *S. oneidensis* is not sufficiently fast. This effect was not observed in the second cycle, where lactate and acetate were both degraded from the beginning. This suggests that the spontaneous mutation happened in all three replicates during the first cycle.

What can be seen quite clearly from the data is coherence between the consumption of acetate and the produced current. Current increases at the point of highest determined acetate concentration and levels out when the acetate concentration in the medium has declined to zero. This implies that the current observed should for the main part be related to acetate consumption by *G. sulfurreducens* and not to the forgoing lactate consumption.

Another possible explanation for the different behaviors in the defined mixed culture might have been intruding oxygen, which would allow *S. oneidensis* to consume lactate as well as acetate in a faster and more efficient way. Extensive intrusion of oxygen due to the MEC used can be excluded here, as the same setup was used for both the defined mixed culture and the pure culture of *G. sulfurreducens*. Extensive leakage of oxygen into the system would have resulted in no growth and no current production by *G. sulfurreducens*. As rather reproducible current profiles were observed with the pure cultures of *G. sulfurreducens*, however, such a leakage can only have happened to a minor extend. However, it is possible that oxygen leaked into reactors in the defined mixed culture when sampling was performed, although it is unlikely that amounts of oxygen vast enough to allow for the observed lactate consumption entered the system during sampling.

The maximum current density of 0.54 ± 0.07 mA/cm^2^ achieved in these experiments was increased by 38% compared to *G. sulfurreducens* pure cultures. This implies the better suitability of mixed cultures as opposed to pure cultures in BESs. It is in accordance with the findings from Prokhorova et al. (2017) who found current densities increased by 32% in mixed culture of *G. sulfurreducens, S. oneidensis* and *G. metallireducens* compared to *G. sulfurreducens* pure cultures. Similarly to the *G. sulfurreducens* pure cultures, CEs achieved with the defined mixed culture were over 100%, indicating viable and highly productive cells and a recycling of hydrogen gas.

CLSM analysis revealed a 35% increased biofilm thickness of the defined mixed culture biofilm compared to the *G. sulfurreducens* pure culture. This value corresponds well to the 38% increased maximum current density observed in the defined mixed culture in the first cycle. CLSM analysis was only conducted after the third cultivation cycle, however additional experiments revealed that the same thickness is already achieved after the first cycle and can therefore be related to the maximum current density seen there. This finding is in accordance with the work of Baudler et al. (2015), who observed an almost linear correlation between different biofilm thicknesses and the respective maximum current densities. Similarly, Zhu et al. (2014) observed a linear correlation between the amount of biomass per electrode surface and the maximum current density.

After current depletion, a medium change was performed removing all planktonic cells and selecting for sessile cells to determine the dependency of the positive effect seen in the defined mixed culture on planktonic *S. oneidensis* cells. The resulting maximum current density was reduced almost 2-fold. In *G. sulfurreducens* pure cultures, this procedure did not lead to decreased maximum current densities. This indicates that the improved current density in the first cycle is caused by planktonic *S. oneidensis* and is not sustained by cells integrated into the biofilm.

During the third chronoamperometric cycle, cytometric analysis was performed with biofilm and planktonic cells of the defined mixed culture, revealing a *G. sulfurreducens* dominated anodic biofilm and a planktonic presence of *S. oneidensis* of 25%. The presence of *S. oneidensis* within the biofilm cannot be verified here, as the amount detected is only insufficiently higher than the falsely positive signal. Thus, the defined mixed culture biofilm should be considered to only contain *G. sulfurreducens*. This is in accordance with the similarity of the electrochemical behavior seen in the defined mixed culture by CV measurements resembling those of *G. sulfurreducens* pure cultures (see Figure S3). Even assuming an amount of approximately 1% *S. oneidensis* cells present in the biofilm, this amount cannot account for the 35% increased thickness of the biofilm. Also, the low current densities seen in the *S. oneidensis* pure culture are likewise not high enough to explain the higher current densities in the defined mixed culture. Thus, from the data presented here, it is clearly the planktonic presence of *S. oneidensis* that has a positive effect on the growth and performance of the *G. sulfurreducens* based biofilm and its current production in the first cycle.

In similar experiments, Dolch et al. (2014) observed an amount of 9% *S. oneidensis* and 91% *G. sulfurreducens* within mixed culture biofilms by quantitative polymerase chain reaction ca. two days after the beginning of experiments. Prokhorova et al. (2017) found *G. sulfurreducens* and *S. oneidensis* at quantities of 95 and 5%, respectively, in anodic biofilms of three-species mixed cultures with *G. metallireducens* after 7 days. However, the long-term behavior of this distribution was not investigated by these authors. The findings presented here suggest that *S. oneidensis* cells are eventually dislodged from the electrode by *G. sulfurreducens*, especially when the supplementation from planktonic *S. oneidensis* cells is hindered. Further, the systems used by these authors were not completely oxygen tight (Dolch et al., 2014; Prokhorova et al., 2017). Traces of oxygen diffusing into the anodic chamber will improve growth of *S. oneidensis* as shown by Teravest et al. (2014). Anaerobic conditions were applied in the present study. Even considering that minor amounts of oxygen might still have leaked into the system, this was not sufficient to sustain the beneficial interactions between *S. oneidensis* and *G. sulfurreducens*.

Nevertheless, both the work of Dolch et al. (2014) and Prokhorova et al. (2017) as well as the work presented here show a positive effect of the combination of *G. sulfurreducens* and *S. oneidensis* in BESs as opposed to respective pure cultures, and there are several possible explanations. *S. oneidensis* has been shown previously to be capable of H_2_ production from lactate (Meshulam-Simon et al., 2007). Correspondingly, Prokhorova et al. (2017) found two hydrogenases in *S. oneidensis* whose expression is upregulated under mixed culture conditions. Thus, H_2_ provided by *S. oneidensis* could function as additional energy source for *G. sulfurreducens* and enhance growth. Lactate can also have been used directly by *G. sulfurreducens* for biofilm formation. However, Speers and Reguera (2012) observed poorer biofilm formation with lactate as electron donor compared to acetate, thus the increased thickness seen here cannot be explained with the different electron donors provided. Further, several prophages in the genome of *S. oneidensis* have been found to lead to cell lysis of a subpopulation of cells, releasing extracellular DNA (eDNA) (Gödeke et al., 2011; Binnenkade et al., 2014). This eDNA was found to be a crucial component in biofilm formation and stability (Gödeke et al., 2011; Binnenkade et al., 2014). Possibly, eDNA released by *S. oneidensis* also has a positive effect on the biofilm formation and stability of *G. sulfurreducens* and can be another explanation for the observed increased biofilm thickness seen in the defined mixed culture here. Finally, *S. oneidensis* secretes flavins (Marsili et al., 2008) which could also be used by *G. sulfurreducens* for enhanced direct electron transfer as was proposed by Okamoto et al. (2014). These authors only showed an enhancement in current of 10%, however, which alone cannot explain the enhanced current generation observed here. Thus, it is probably a combination of the above discussed effects that create the beneficial circumstances for *G. sulfurreducens* in the defined mixed culture.

The aim of the present comparative study was to investigate beneficial interactions in defined mixed cultures of *G. sulfurreducens* and *S. oneidensis* compared to respective pure cultures and to test the stability of the incorporation of *S. oneidensis* in the electrochemically active biofilm in an anaerobically operated MEC setup. Observations by others who reported that defined mixed cultures of *G. sulfurreducens* and *S. oneidensis* perform better in BESs as opposed to the individual pure cultures could be confirmed (Dolch et al., 2014; Prokhorova et al., 2017). However, the stable incorporation of *S. oneidensis* into the biofilm could not be confirmed here. Only the planktonic presence of *S. oneidensis* had a positive effect on *G. sulfurreducens* and lead to thicker *G. sulfurreducens* based anodic biofilms, accompanied by an increased current density. A correlation between biofilm thickness and maximum current production could therefore be found, confirming others' findings (Zhu et al., 2014; Baudler et al., 2015).

The findings have implications regarding the application of defined mixed cultures in continuous systems operated for longer time periods. If the positive effect of mixed cultures is to be sustained, a stable integration of *S. oneidensis* or the retaining of planktonic *S. oneidensis* cells is necessary. In a continuously operated flow cell, cell washout would occur; therefore rather a continuously operated bioreactor is advantageous with a flow rate below the maximum growth rate of *S. oneidensis*. It also appears meaningful to optimize cultivation conditions for *S. oneidensis* to ensure its sustained presence in the mixed culture. This could be achieved by exerting a controlled introduction of oxygen to facilitate growth of *S. oneidensis*. It has been shown by Teravest et al. (2014) that micro-aerobic cultivation conditions in contrast to anaerobic conditions lead to an increased biofilm thickness and amount of planktonic cells in MECs operated with *S. oneidensis*, concomitant with higher current densities. Since this effect is due to the consumption of oxygen by *S. oneidensis, G. sulfurreducens* would not be affected by oxygen intrusion and the increased planktonic amount of *S. oneidensis* might instead lead to an improved overall electrochemical performance of the defined mixed culture.

## Data Availability

Flow repository data is available via the following http://flowrepository.org/id/RvFrXnk3EuaeLev2LtY5uhRkpIUMSuTc9WilOMQ6LRJqmDWZj4fyR4hvpSnNYKpJ.

## Author Contributions

CE, KD, and RK conceived the study. CE conducted the main lab work, all electrochemical experiments and CLSM analysis and the literature review. FS and SM performed flow cytometry analysis. CE and FS prepared the manuscript. KD, RK, and US supervised the study, participated in its design and coordination and helped to draft and revise the manuscript. All authors read and approved the final manuscript.

### Conflict of Interest Statement

The authors declare that the research was conducted in the absence of any commercial or financial relationships that could be construed as a potential conflict of interest.
