# Supplementary material for: Long-Term Behavior of Defined Mixed Cultures of Geobacter sulfurreducens and Shewanella oneidensis in Bioelectrochemical Systems
Source: Front Bioeng Biotechnol. 2019 Mar 27;7:60. doi: 10.3389/fbioe.2019.00060 (PMC6445848; doi:10.3389/fbioe.2019.00060)
Supplement: Supplementary file 1 [file Data_Sheet_1.PDF]

Supplementary material

Cytometric analysis

The parent gate for cytometric analysis that was created with Summit Ver. 4.3 and comprises all stained cells excluding noise and beads is depicted in **Fig. S1**. Further, the cell gate bearing to the parent gate that was defined in FlowJo Ver. 10 is shown with an exemplary sample in **Fig. S2**.

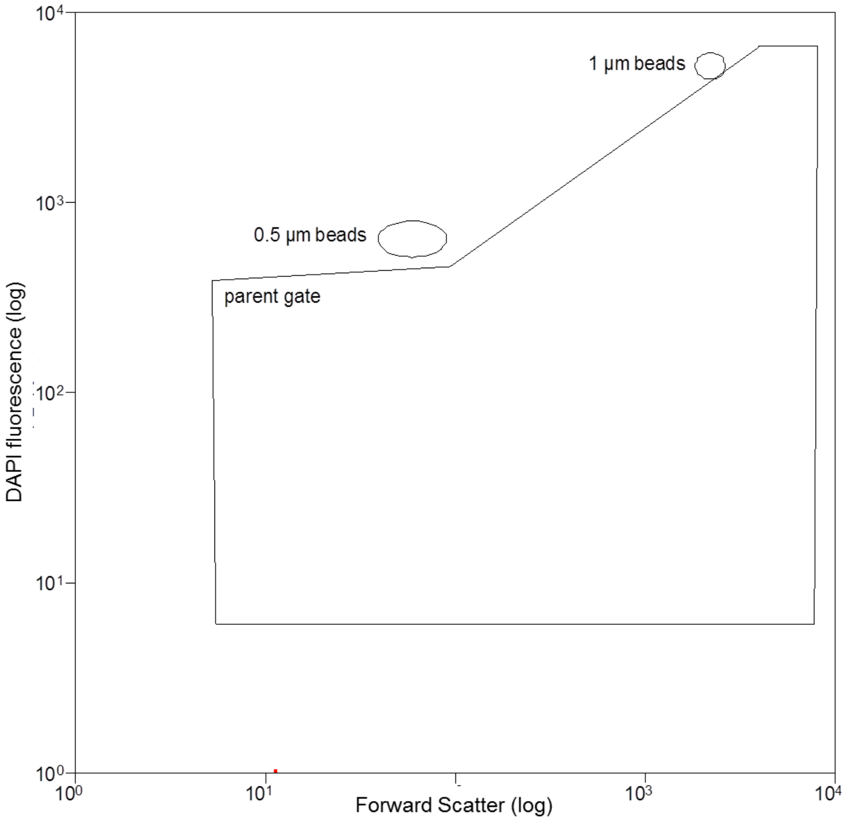

Figure S1: Parent gate for flow cytometry analysis comprising all stained cells and excluding noise and beads as created with Summit Ver. 4.3.

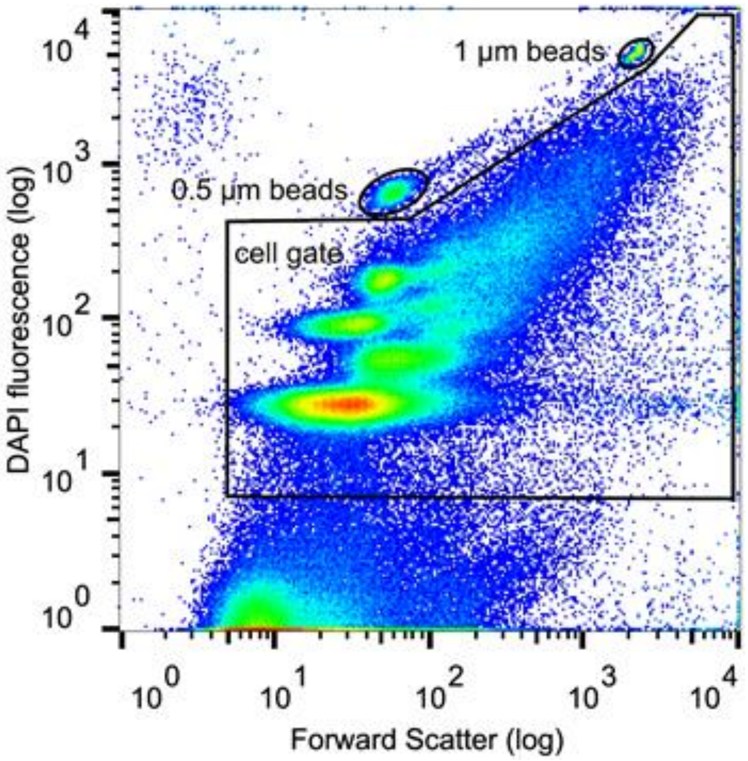

Figure S2: Exemplary sample of cell gate bearing to the parent gate defined in FlowJo Ver. 10.

Biofilm characterisation via cyclic voltammetry analysis

During cultivation cycles under turnover conditions, cyclic voltammetry measurements were performed for the determination of the formal potential of redox components within the biofilm actively participating in current production. Cyclic voltammograms (CVs) were recorded at a scan rate of 0.25 mV/s between 0.2 and -0,45 $V_{Ag/AgCl}$ in case of the pure cultures and between 0.6 and -0.45 $V_{Ag/AgCl}$ in case of the defined mixed culture. Three repetitions were done for each CV. Exemplary CVs of *G. sulfurreducens* pure cultures, *S. oneidensis* pure cultures and *G. sulfurreducens* / *S. oneidensis* defined mixed cultures are depicted in **Fig. S3**.

CVs from defined mixed cultures strongly resemble those of *G. sulfurreducens* pure cultures. In contrast to this, in the *S. oneidensis* pure culture CV the electrochemical signal discernable is not much more substantial than the capacitive current. From the point of inflection in the CV curves, the formal redox potential of the redox components responsible for the electron transfer to the anode can be determined. For the defined mixed culture and the *G. sulfurreducens* pure culture this potential is at -0.32 ± 0.02 $V_{Ag/AgCl}$ and -0.319 ± 0.011 $V_{Ag/AgCl}$ respectively, so at exactly the same point. This indicates that *G. sulfurreducens* is the main current producing organism within the defined mixed culture, which is in accordance with the non-detectably low amount of *S. oneidensis* cells in the biofilm. It should be noted that the formal potentials determined here are most likely an average signal from different redox active compounds with redox potentials around -0.32 $V_{Ag/AgCl}$, as more than one active redox centre is participating in electron transfer (Fricke et al. 2008).

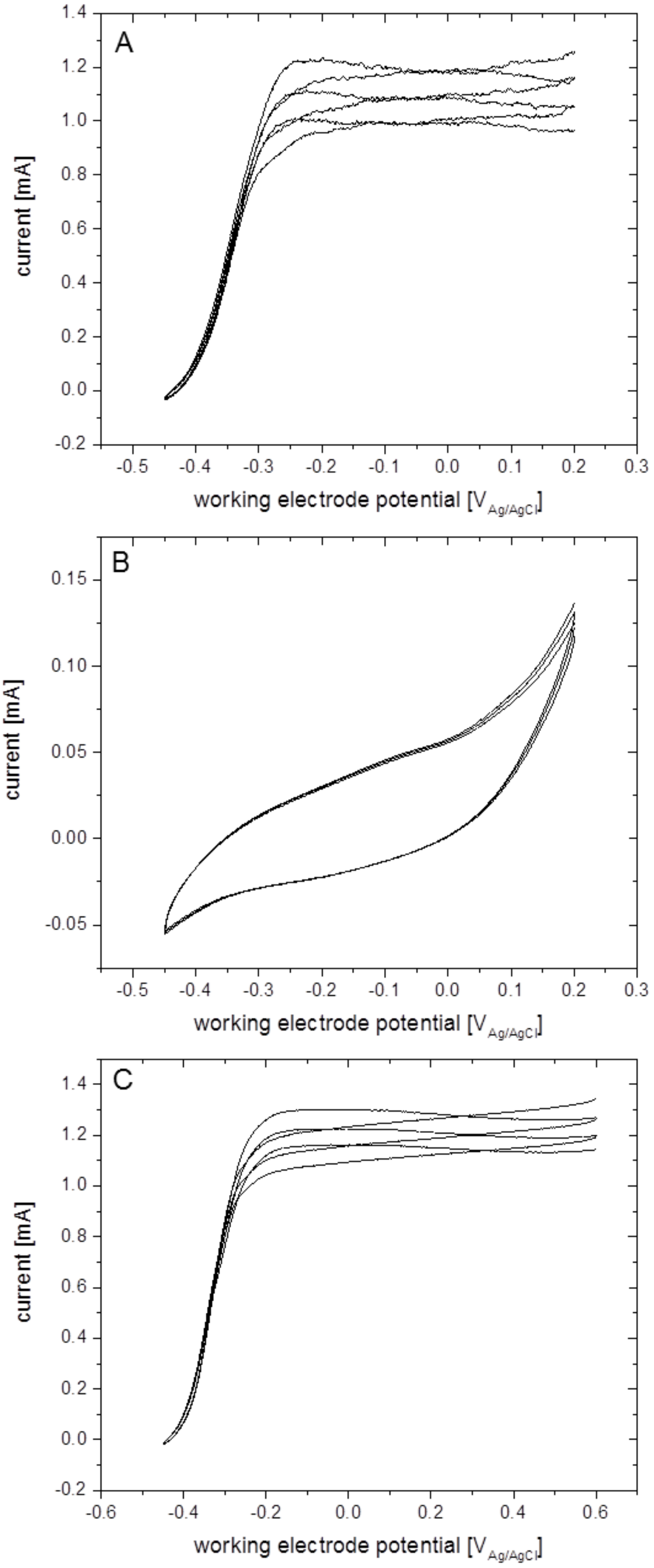

Figure S3: Exemplary cyclic voltammograms (three repetitions) under turnover conditions from (A) a *G. sulfurreducens* pure culture, (B) an *S. oneidensis* pure culture and (C) a defined mixed culture at a scan rate of 0.25 mV/s. Cyclic voltammograms were recorded during the second cycle of experiments.
